# Supplementary material for: Caveats to Deep Learning Approaches to RNA Secondary Structure Prediction
Source: Front Bioinform. 2022 Jul 11;2:835422. doi: 10.3389/fbinf.2022.835422 (PMC9580944; doi:10.3389/fbinf.2022.835422)
Supplement: Supplementary file 1 [file DataSheet1.PDF]

Supplementary Material

1 SUPPLEMENTARY DATA

The ML-modles and the trainingsdata can be found on github `https://github.com/ViennaRNA/RNAdeep`

2 SUPPLEMENTARY TABLES AND FIGURES

2.1 Figures

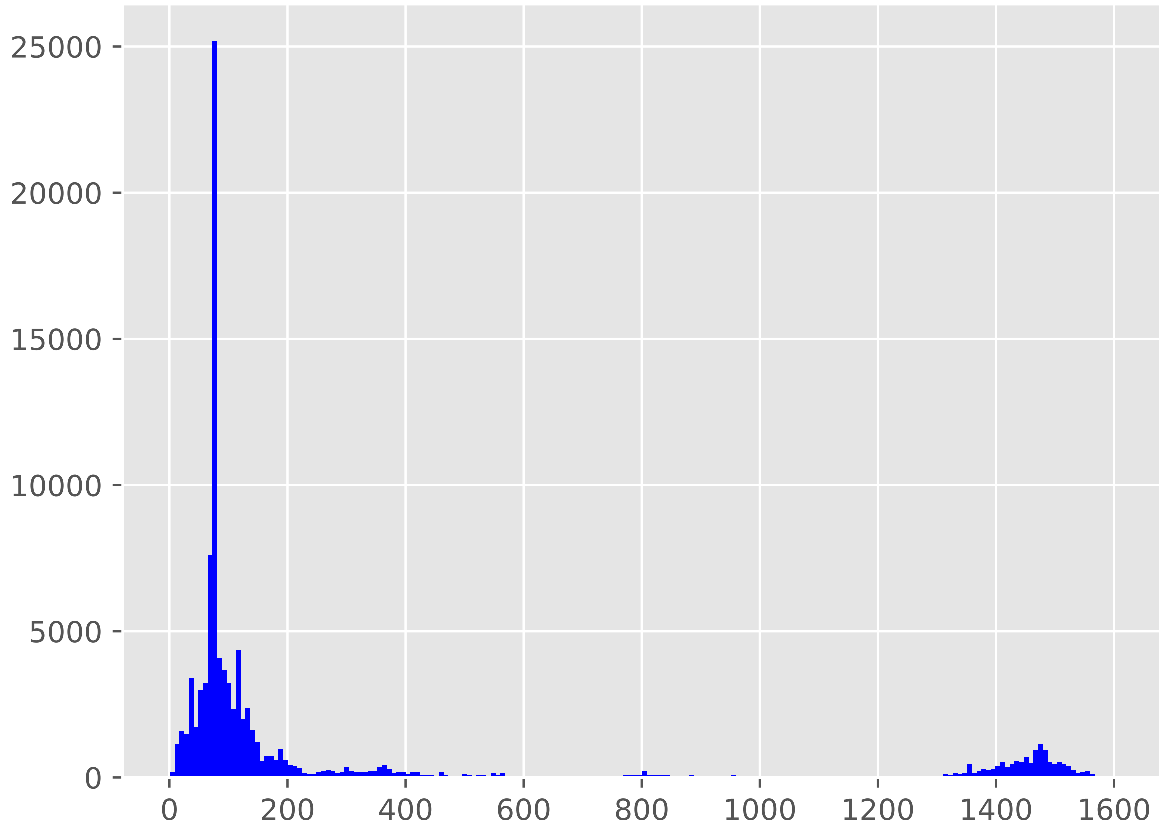

Figure S1. Length distribution of the sequences in the bpRNA-1m dataset Version 1 `http://bprna.cgrb.oregonstate.edu/`. The highest peak correspond to tRNAs of a length of about 75 nucleotides (nts). For the plot the dataset was truncated at length 1600 nts (removing 736 sequences longer than 1600 nts).

REFERENCES

Singh, J., Hanson, J., Paliwal, K., and Zhou, Y. (2019). RNA secondary structure prediction using an ensemble of two-dimensional deep neural networks and transfer learning. *Nat Commun* 10, 5407.

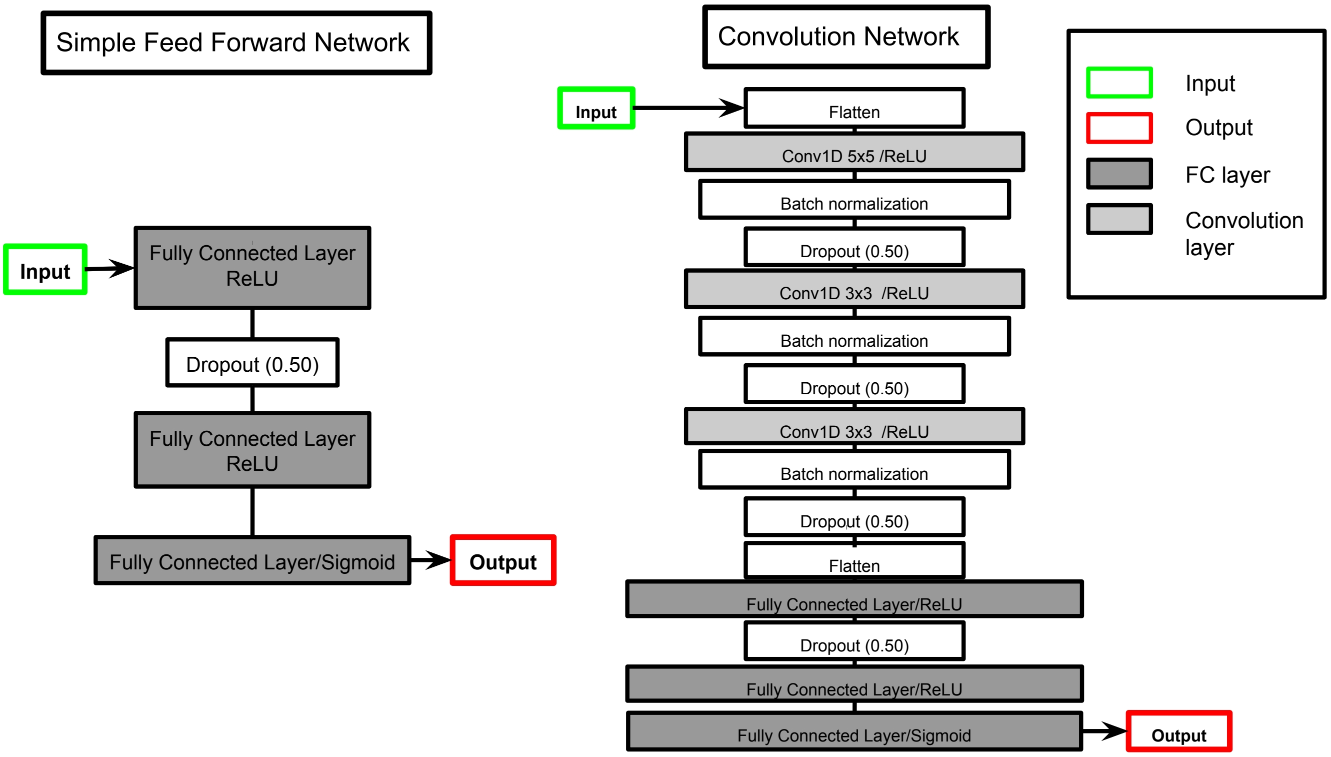

Figure S2. Neural network architectures for the sliding window approach. (left) feed forward neural (FFN) network (right) 1D convolutional neural network (CNN)

doi:10.1038/s41467-019-13395-9

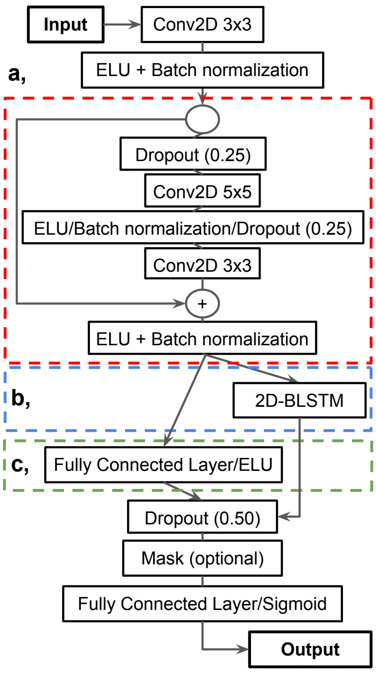

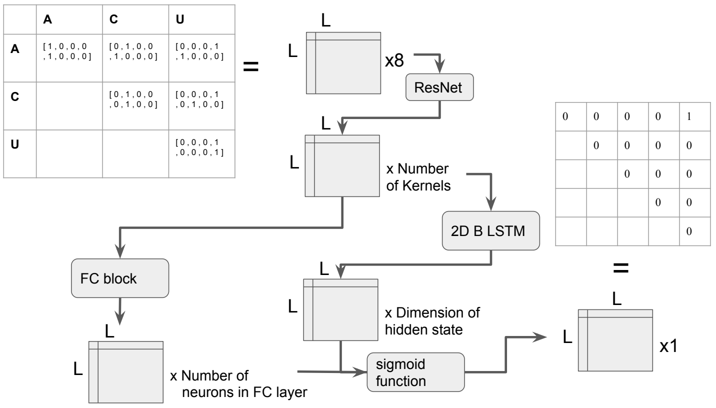

Model	Filters	ResNet blocks	LSTM blocks	LSTM neurons	FC blocks	FC neurons
0	64	20	0	0	2	512
1	48	16	0	0	2	512
3	64	30	1	200	0	0

Figure S3. Detailed view of the reimiplemented network architectures of the SPOT-RNA network (Singh et al., 2019) refered to as model 0-3. The repeat numbers for the ResNet (in red), the LSTM (in blue) and the fully connected layers (in green) are listed in table on the bottom right. How the data is fed into and retreved from the models is shown in the schematic figure on the top right.

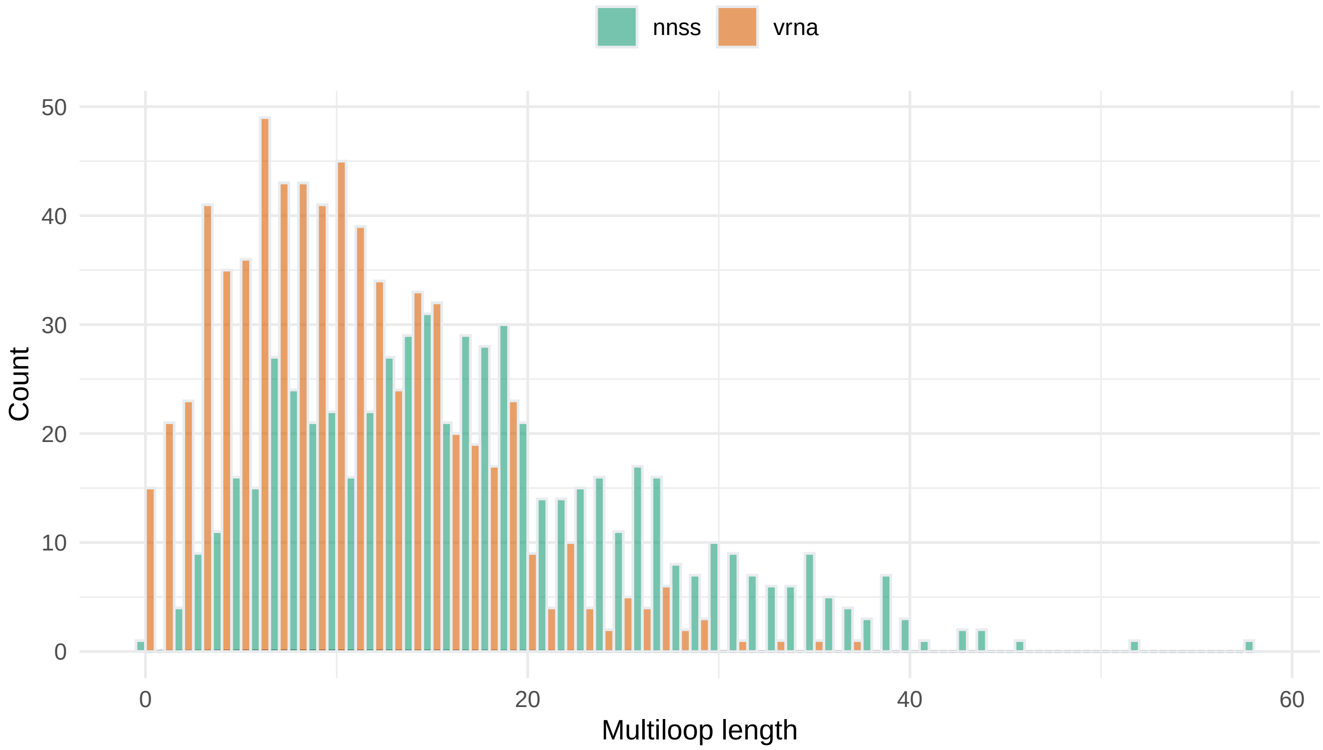

Figure S4. Length distribution of multi-loops of the validation set of length 100 nt predicted by the neural network (nnss) and `ViennaRNA` (vrna), respectively.
